# Primary retroperitoneal malignant melanoma

**Published:** 2012-06-04

**Authors:** Aziz Zentar, Rabie Makhmari, Hicham Elkaoui, Mohammed Bouchentouf, Hicham Lahnini, Said Belhamid, Ahmed Bounaim, Abdelmounaim Aitali, Khalid Sair

**Affiliations:** 1 Mohammed V Rabat, Maroc

**Keywords:** Melanoma, malignant, primary retroperitoneal

## Abstract

We report a case of an extremely rare primary malignant melanoma presenting in the retroperitomeum, indurcing a diagnostic and management problem.

## Introduction

Malignant melanoma is a tumour arising from the melancytes which are derived from the neural crest, it migrate during the embryological development and may be found in the non cutoneons site. With a predisposition to the possibility of developing melanoma in adult life [1]. In clinical practice and medical literature, malignant melanoma usually appears in typical sites where melanocytes can be found (skin, eyes, meninges and anal region) [2].Malignant melanoma of the skin is easily diagnosed, but some, particulary those presenting as non cutaneous primaries or as metastatic disease may closely mimic other tumours [3]. Malignant melanoma of unknown origin accounts for 5-10% of melanoma cases [4]. Primary retroperitoneal tumors are rare and most of the time malignant, because of the large space where they grow, they are often discovered lately as they are large [5]. Malignant melanoma representing in the retroperitoneum are usually metastatic in patients with a history of melanoma [5, 6]. Primary retroperitoneal malignant melanoma is extremelly rare and only a few descriptions of this presentation have been found in the medical literature only 6 cases. The present report describes a new case of primary retroperitoneal malignant melanoma, indurcing a diagnostic and management problem.

## Patient and case report

**Figure 1:**
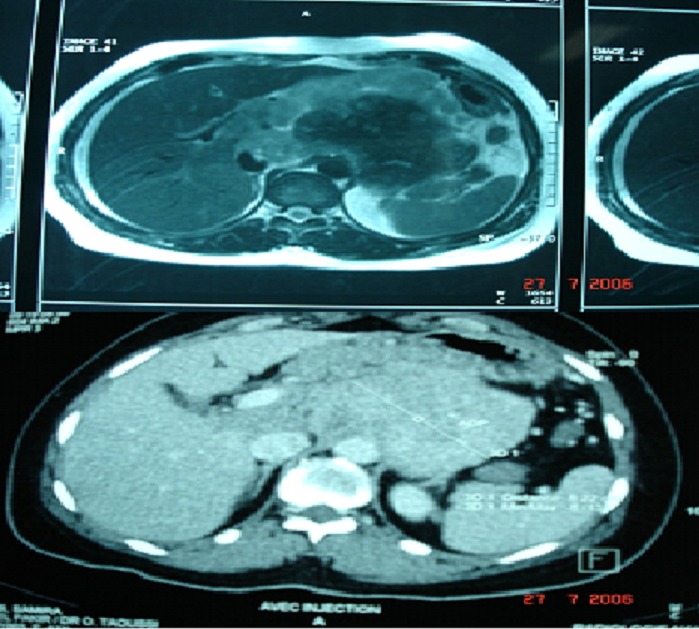
Abdominal CT scan: A large retroperitoneal tumor measuring 8, 22x8, 15 cm, heterogeneous, displaced the pancreas, second and third duodenum. The mass was also seen to be protruding into the retro aortic region. No liver metastatic

**Figure 2:**
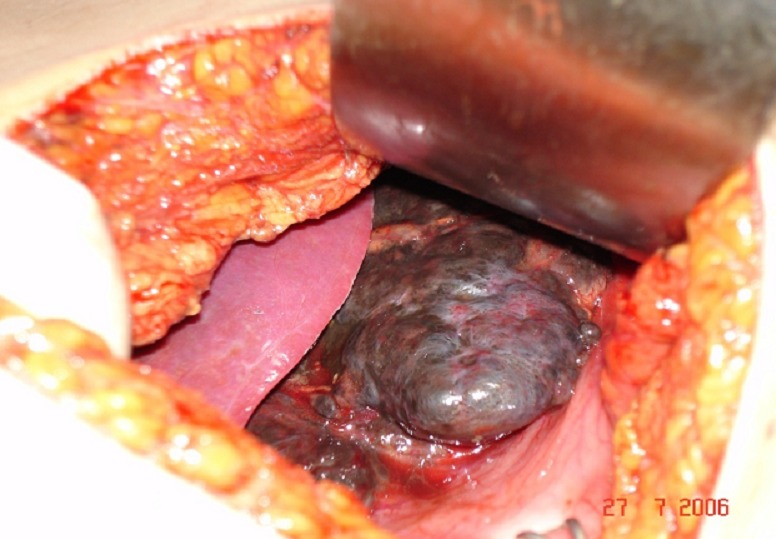
Surgical exploration: A large retroperitoneal tumour, blackish with grooved surface and diameter of approximately 14 cm, which was displacing and encompassing aorta, inferior vena cave and left ureter, no liver metastatic

**Figure 3:**
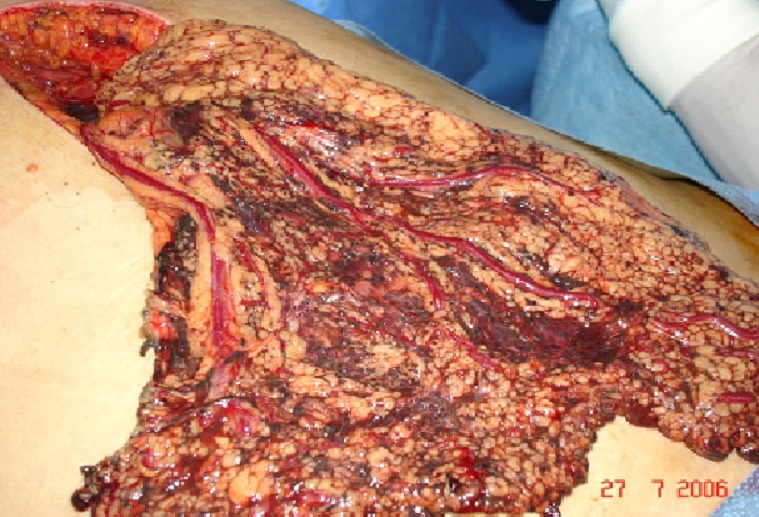
The greater omentum: The seats of multiple solid blackish lesions were suspected metastatic lesions

We report a 34-year-old woman hospitalised with a 3-month history of recurrent abdominal pain, abdominal distension, asthenia, anorexia, followed by fever and vomiting since. She had no personal history of medical disease especially cutaneous or ocular melanoma. On admission the patient was pale, in her physical examination, pallid skin and mucous membranes were observed. Her abdomen was soft and yielding and tender to deep palpation of the left hypochondrium, where a masse could be felt. The rest of physical examination was normal. In the medical tests carried out the only altered parameters were hemoglobin 10 g/dl, C reactive protein (CRP) 20mg/l and blood glucose 141mg/dl. The liver function test showed values which within normal limits. An abdominal ultrasound revealed a mass with diameter of approximately 9 cm at area between the pancreas, the left adrenal gland and upper pole of the left kidney. An abdominal CT scan confirmed the presence of a large retroperitoneal tumor measuring 8, 22x8, 15 cm, heterogeneous, displaced the pancreas, second and third duodenum. The mass was also seen to be protruding into the retro aortic region (Figure 1). We decided to perform surgery with the patient undergeneral anesthesia. We made a midline suprainfraumbilical incision and found a large retroperitoneal tumour in left hypochondrium, blackish with grooved surface and diameter of approximately 14 cm, which was displacing and encompassing aorta, inferior vena cave and left ureter, no liver metastatic (Figure 2). The greater omentum was the seat of multiple solid, blackish, lesions were suspected metastatic lesions (Figure 3). We decided to perform a simple biopsy of huge tumor, and omentectomy. The histopathology report; the tumour cells are mostly spindle shaped, pleomorphic hyperchromatic nuclei, binucleate, with many cells showing intracytoplasmic melanin pigment. Immunohistochemical staining revealed strong, diffuse positive S-100 protein and staining for the melanoma markers HMB45 and Milan A, was suggestive of malignant melanoma, and omentum’s lesion metastatic. Palliative Chemotherapy with Interferon alpha 2b (IFN∞2b) was begun by the oncology service, and she died 4-months later. The detailed clinical history showed that there was no previous melanoma which was excised or regressed and that the patient did not have any lesions on the skin or in the eyes. So, the case was diagnosed as primary retroperitoneal malignant melanoma.

## Discussion

Primary malignant melanoma is most frequently found on the skin and which, on very rare occasions, may originate in the eye (choroid plexus). Melanomas have also been found in the month cavity, larynx and bronchi, the oesophagus, the rectum, the genitourinary system, the meninges, the ovaires, the uterine cervix, the vagina and adrenal glands [7]. Malignant melanoma of unknown origin accounts for 5-10% of the melanoma cases [4]. Whenever melanoma is found in the gastrointestinal tract or in any other retroperitoneal site, a primary cutaneous lesion is nearly always documented. It is rarely absent, as in the present case. Our patient did not have skin lesion, did have any relevant past of history of melanoma [6]. One of the major obstacles for clinicians is identifying whether the tumor is really a primary tumor or whether it is metastatic [7]. Generally, the sign and symptoms that characterize these clinical entities are not at all specific, with pain being the most common manifestation, together with imprecise gastrointestinal disorders, caused by the compression of the structures adjacent to these neoplasies [7], in the present case, the patient presented with a huge retroperitoneal masse, with imprecise gastrointestinal symptoms which not suggested a primary retroperitoneal tumor. Primary retroperitoneal tumors are rare and most of the time malignant, because of the large space where they grow, they are often discovered lately as they are large [5], they are often sarcomas (0,5-1%) or tumours arising from adrenal gland [5–8]. The present case was initially suspected as sarcoma on the basis of the CT scan findings, as the rest of the retroperitoneal tissues, the adrenal gland and kidney were within normal limits. Malignant melanomas show many variations in the cytomorpholigical architecture and the stromal components [3–9]. Immunohistolchemical study leads to the correct diagnosis [7]. In the present case, immunohistochemical staining revealed diffuse positive S-100 protein and staining for the melanoma markers HMB45 and Milan A. The clinical history showed that there was no previous melanoma which was excised or regressed and that the patient did not have any lesions on the skin or in the eyes. So, the case was diagnosed as primary retroperitoneal malignant melanoma. Clear guidelines for the therapy of primary retroperitoneal malignant melanoma have not been settled. This mainly results from the rarety of this tumour and to the difficulty in collecting a consistent number of cases in a homogeneous and rational way [10]. Interferon-∞ is the first substance in adjuvant therapy of melanoma which has shown a significant improvement of disease-free survival and in some prospective randomised trials, of overall survival. There are, however, conflicting results from different clinical trials [9]. The patient died 4-month after the beginning of palliative chemotherapy with IFN∞2b.

## Conclusion

Although malignant melanoma is usually described as a metastatic lesion, in this case because another primary site of melanoma was not identified the present case can be considered a primary retroperitoneal malignant melanoma. This is a rare but aggressive disease, often associated with metastatic spread at presentation.
